# Supplementary material for: The interactome of intact mitochondria by cross-linking mass spectrometry provides evidence for coexisting respiratory supercomplexes
Source: Mol Cell Proteomics. 2017 Dec 8;17(2):216–32. doi: 10.1074/mcp.RA117.000470 (PMC5795388; doi:10.1074/mcp.RA117.000470)
Supplement: Supplemental Data [file supp_RA117.000470_133922_0_supp_23978_fzffwf.zip › spectra_annotation/mito_spectra_annotation/381-1-1-1-12-1.pdf]

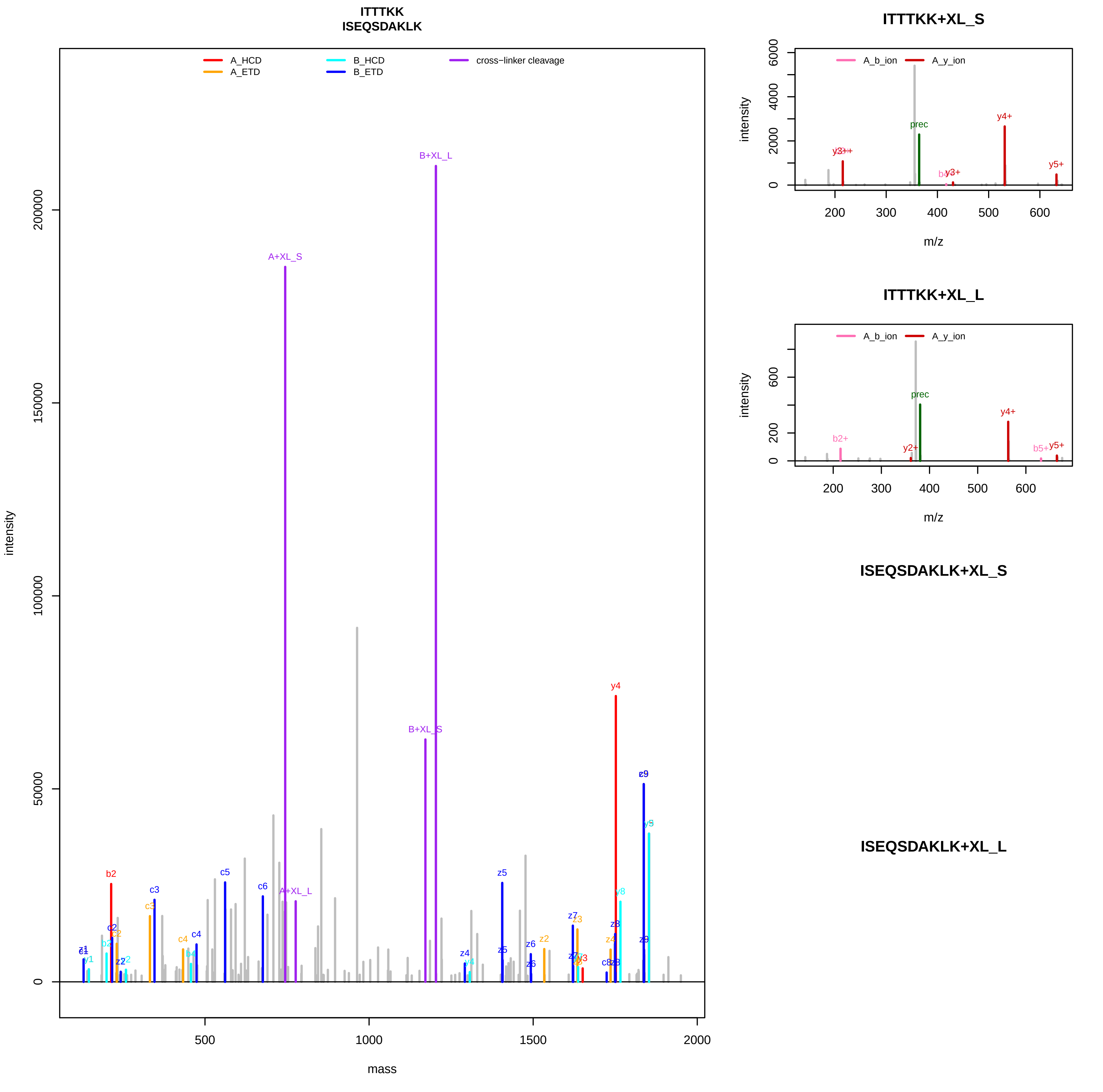

ITTTKK
ISEQSDAKLK
A_HCD
A_ETD
B_HCD
B_ETD
cross−linker cleavage
B+XL_L
A+XL_S
B+XL_S
A+XL_L
y4
z9
y5
b2
c3
c5
c6
z5
z7
z3
y8
z8
z2
z4
c4
c2
intensity
mass
ITTTKK+XL_S
A_b_ion
A_y_ion
prec
y4+
y5+
y3+
m/z
ITTTKK+XL_L
b2+
y2+
b5+
ISEQSDAKLK+XL_S
ISEQSDAKLK+XL_L
